# Heterochromatin variation among the populations of *Mus terricolor* Blyth, 1851 (Rodentia, Muridae) chromosome type I

**DOI:** 10.3897/CompCytogen.v7i2.4136

**Published:** 2013-05-28

**Authors:** Mahua Rudra, Min Bahadur

**Affiliations:** 1 Genetics and Molecular Biology Laboratory, Department of Zoology, University of North Bengal, Siliguri-734013, West Bengal, India

**Keywords:** Heterochromatin, C-banding, *Mus terricolor*

## Abstract

Twenty five to thirty specimens each from ten populations of *Mus terricolor* of the Terai and the Dooars regions of the Darjeeling foothills of West Bengal were cytogenetically analyzed using C-banding. Results showed intra- and inter- population variation of C-band positive heterochromatin ranging from very large blocks to minute amounts or even complete absence of heterochromatin. Large blocks of centromeric C-bands were found in Bidhan Nagar, Garidhura, Malbazar, Nagrakata and Maynaguri populations in most of the autosomes, while the rest of the populations had large blocks of C-bands on a few autosomes only. Such intra- and inter- population variation may be due to accumulation of C-positive heterochromatin, which has not got fixed homogeneously in all autosome pairs. X-chromosomes invariably possess a C-banded short arm a telomeric C-band at the distal end of the long arm in all populations. The entire Y-chromosome was C-band positive with slight population differences in staining intensity. The results suggest quantitative as well as qualitative variation of C-positive heterochromatin.

## Introduction

The earth-colored mouse *Mus terricolor* is a common field mouse of the Indian sub-continent infesting paddy and wheat fields and was known as *Mus dunni* Wroughton, 1912 until Musser and Carleton (1993) synonymised it with the former name. This *Mus* species co-exists in the same habitat with the sibling species *Mus booduga* Gray, 1837. Both the species have 2n=40 chromosomes like other species of the subgenus *Mus*. In contrast to all acrocentric chromosomes of *Mus booduga*, *Mus terricolor* is characterized by a large submetacentric X and a large acrocentric Y chromosome (Matthey and Petter 1968, Sharma and Garg 1975, Markvong et al. 1975, Manjunatha and Aswathanarayana 1979). The short arm of X and the entire Y chromosome are heterochromatic (Sharma et al. 1986, 1990). Compared to the conserved karyotype of *Mus booduga* throughout its distribution range, *Mus terricolor* shows divergent karyotypes due to a variable number of heterochromatic short arms established in homozygous condition (Sen and Sharma 1983, Sharma et al. 1986, 1990) which indicates that *Mus terricolor* is in an active phase of evolutionary differentiation. Cytogenetic studies by Sharma and his associates revealed that *Mus terricolor* is differentiated into three distinct karyotypes (2n = 40) designated as chromosome types I, II and III. Chromosome type I has a wide distribution throughout the subcontinent except the southern peninsular region and has all acrocentric autosomes with C-band positive minute perceptible short arms. Chromosome type II, characterized by two pairs of submetacentric autosomes 1 and 3 with heterochromatic short arms, is found in Mysore and Erode in the peninsular India, while Chromosome type III distributed in Chennai, Tirupati and Madurai is characterized by three pairs of submetacentric autosomes 1, 3 and 6 with heterochromatic short arms.

Karyotype differentiation in *Mus terricolor* is due to acquisition of varying amount of constitutive heterochromatin in and around the centromere on specific autosomes. Different studies have been carried out in *Mus terricolor* chromosome types and their populations covering vast regions of southern, central and western part of India (Sharma and Garg 1975, Sen and Sharma 1983, Sharma et al. 1986, Sharma 1996) but populations from West Bengal in eastern India were not included. The northern part of West Bengal, characterized by hills (Darjeeling District) and the Terai and the Dooars regions in the foothills, is well known for biodiversity and diverse ecological features.

In view of the aforesaid situation, this study has been conducted to know the extent of intra- and inter- population heterochromatin variation in *Mus terricolor* chromosome type I from the Terai and the Dooars regions of foothills of Darjeeling in West Bengal.

## Materials and methods

### Animals

The individuals of *Mus terricolor* were collected from paddy fields by digging burrows during harvesting season of the crop (November to December) from ten different locations of the Terai and the Dooars of foothills of Darjeeling in West Bengal, India. Three of the collection sites are in the Terai and seven collection sites are in the Dooars. The river Tista separates the Terai and the Dooars as a physical barrier. The name of the collection sites and their provisional geographical coordinates has been shown in Table 1 along with population name and number of individuals studied from each site. Animals were collected and identified by mitotic chromosome preparation. 25–30 individuals from each population were analyzed for this study. Individuals of *Mus terricolor* are abbreviated for convenience according to their collection localities. In the Terai region these are NXL (Naxalbari), GDH (Garidhura), BDN (Bidhan Nagar), and in the Dooars these are APD (Alipurduar), RBD (Rohimabad), KGM (Kumargram), MNG (Maynaguri), NGK (Nagrakata), MLB (Malbazar) and CBH (Cooch Behar).

**Table 1.** Populations, collection sites, geographical coordinates and number of studied individuals of *Mus terricolor*.

**Populations**	**Collection sites**	**Geographical coordinates**	**No. of specimens**
	**Terai region**		
NXL	Naxalbari	26°41'00"N, 88°13'00"E	30
GDH	Garidhura	26°48'24"N, 88°16'38"E	28
BDN	Bidhan Nagar	26°16'00"N, 88°12'00"E	28
	**Dooars region**		
APD	Alipurduar	26°31'21"N, 89°32'37"E	25
RBD	Rohimabad	27°54'00"N, 80°30'05"E	27
KGM	Kumargram	26°36'50"N, 89°49'30"E	29
MNG	Maynaguri	26°33'07"N, 88°49'26"E	25
NGK	Nagrakata	26°54'00"N, 88°50'00"E	29
MLB	Malbazar	27°01'00"N, 89°20'17"E	30
CBH	Cooch Behar	26°32'05"N, 89°07'12"E	26

### Mitotic Chromosome Preparation

Mitotic chromosomes were prepared from bone marrow of colchicine injected mice with hypotonic treatment following air dried method after Lee and Elder (1980) and modified by Baker et al. (1982).

### C-Banding

C-banding was carried out using the BSG (Barium/Saline/Giemsa) method of Sumner (1972) with slight modifications. Two to three day old slides were treated with 0.2N Hydrochloric acid for 1h at room temperature followed by 2-3 rinses in distilled water. The slides were treated in freshly prepared 5% aqueous solution of Barium hydroxide [Ba(OH)_2_] at 50°C for about 2-5 minutes, followed by thorough rinsing in distilled water.

Slides were dried and incubated for 2 h at 60°C in 2 x SSC, pH 7.2 (0.3M Sodium Chloride containing 0.03 M Tri-Sodium Citrate). SSC treated slides were rinsed in distilled water and stained in 5% Giemsa, buffered with phosphate buffer (pH 6.8) for 20–30 minutes and were differentiated in distilled water, dried and mounted in DPX medium.

### Karyotype preparation

A minimum of 10 plates of metaphase spreads were scored for each specimen and karyotypes were prepared from selected metaphase plates. The chromosomes were numbered on the basis of euchromatic long arms as per recommendations of the Committee on Standardized Genetic Nomenclature for mice (1972).

## Results

**Figure 1.**
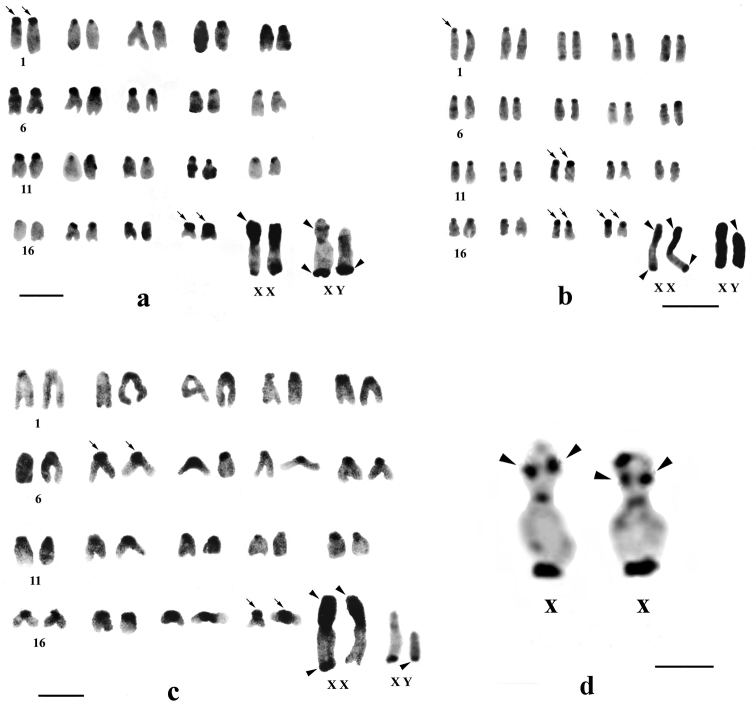
C-banded karyotypes of *Mus terricolor* type I from Terai populations. **a** NXL **b** BDN **c** GDH population **d** segmental C-bandon short arm of X chromosome in *Mus terricolor* from NXL population. Centromeric C-bands are thin arrowed, C-band in short arms of X, entire Y and telomeres of X and Y are arrow headed. Bar = 4µm.

**Figure 2.**
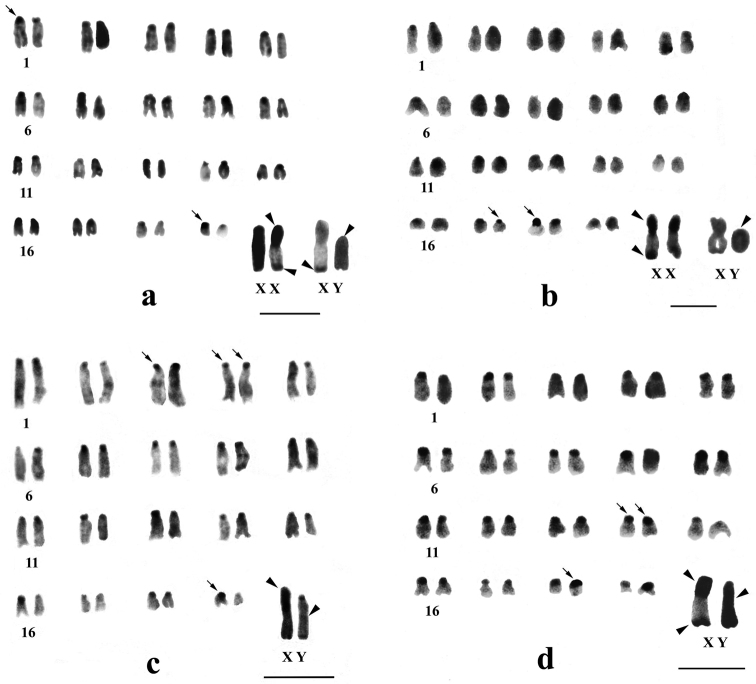
C-banded karyotypes of *Mus terricolor* type I from Dooars populations. **a** APD **b** RBD **c** KGM **d** MNG populations. Centromeric C-bands are thin arrowed, C-band in short arms of X, entire Y and telomeres of X and Y are arrow headed. Bar = 4µm.

**Figure 3.**
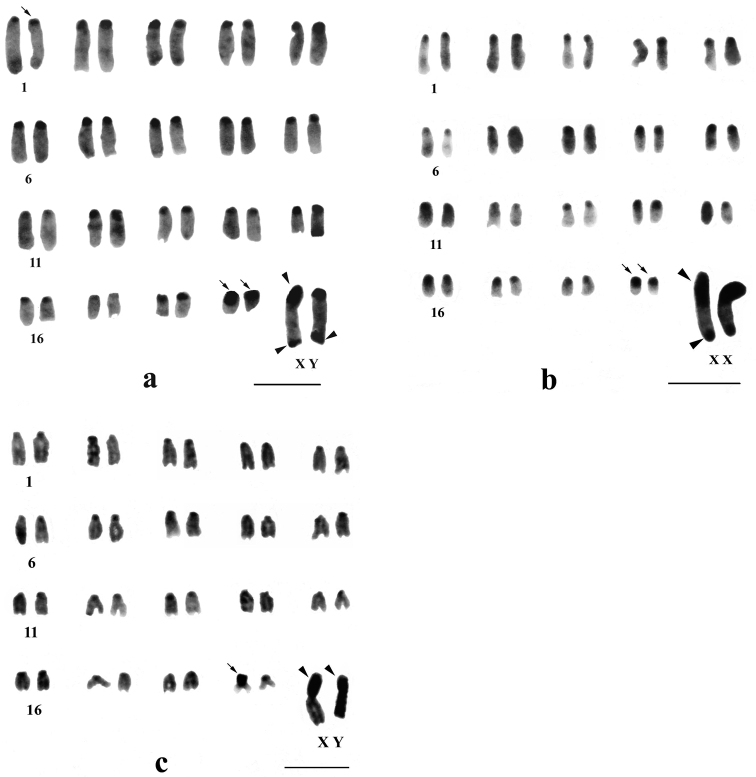
C-banded karyotypes of *Mus terricolor* type I from Dooars populations. **a** MLB **b** NGK **c** CBH populations. Centromeric C-bands are thin arrowed, C-band in short arms of X, entire Y and telomeres of X and Y are arrow headed. Bar = 4µm.

All the individuals of *Mus terricolor* analyzed from ten populations of the Terai and the Dooars demonstrated the diploid number 2n=40 with all acrocentric autosomes and a large submetacentric X and a large acrocentric Y chromosomes in the complement as characteristic. No chromosomal polymorphisms like inversion and Robertsonian translocations were observed. Chromosomes prepared from each individual showed C-band staining, however, few metaphases in each slide either did not show C-band staining or has weak stain. Analyzable metaphase spreads always showed C-bands shown in the representative karyotypes from each population (Figs 1–3).

### Autosomal heterochromatin variation

Differential C-banding revealed extensive heterochromatin variation between and within populations. C-bands were found to be localized in the centromeric region of autosomes throughout the populations varying in size. According to the size of C-bands, the heterochromatin was divided into large blocks, and small to minute C-positive heterochromatin (Table 2). Results showed that individuals of BDN, GDH, MLB, NGK and MNG had large blocks of centromeric heterochromatin in most of the autosomes (Figs 1b, c, 2d, 3a, b). Moreover, the distribution of C-positive heterochromatin was not found to be homogeneous in all autosome pairs. Each chromosome of such pair was stained differentially. Autosome pair 19 consistently showed a large block of C-positive heterochromatin in almost all populations of the Terai and the Dooars with variation between the homologs of the pair (Figs 1–3). In the individuals of populations GDH, BDN, RBD, MLB, NGK and MNG large blocks of heterochromatin were also observed in chromosome 18, which was fixed in homologous condition. In contrast to other populations, NXL, RBD, APD, KGM and CBH were found to have few autosomes with prominent large blocks of C-bands (Figs 1a, 2a,b, c, 3c). Interestingly, autosome 16 was found to be C-band negative in *Mus terricolor* NXL of the Terai while rest of the autosomes showed moderate to prominent C-bands (Fig. 1a).

**Table 2.** C-band variation in different populations of *Mus terricolor*. (s)-Heterogeneity of C-band between homologous autosome pair; SA-short arm of X; LA- long arm of X; WA- entire Y; + denotes intensity of C-band staining.

**Population**	**Size and location of C-positive heterochromatin in autosomes**		**C-positive heterochromatin in sex chromosomes**		
	**Centromere**		**X**		**Y**
	**Large**	**Small to Minute**	**SA**	**LA**	**WA**
NXL	1,4-8,11, 19	2,3,9,10,12-15,17,18	+++	+	+
GDH	2(s),3(s), 4(s),5-8, 10-13, 15-19	1,2(s),3(s),4(s),9, 14	+++	+	++
BDN	1-14,17(s),18,19	15,16,17(s)	+++	+	+++
APD	17(s),19(s)	1-16,17(s),18,19(s)	+++	+	++
RBD	5(s),7,10,11(s),12(s),16,18,19	1-4,5(s),6,8,9,13-15,17	+++	+	+++
KGM	17(s),19(s)	1-16,17(s),18,19(s)	+++	+	++
MLB	2-10,12-15,18,19	1,11,16,17	+++	+	++
NGK	2,4,5,8-11,14,16-19	1,3,6,7,12,13,15	+++	+	_
MNG	1-14, 16,18,19	15,17	+++	+	+++
CBH	2,13,16,19	1,3-12,14,15, 17, 18	+++	+	+++

### Heterochromatin variation in sex chromosomes

The X and Y chromosomes of *Mus terricolor* were found to be consistently C-band positive in all populations, however, minute differences were observed in size and intensity of C-bands both at intra- and inter- population level (Table 2, Figs 1–3).

### X chromosome

The short arm of X chromosomes in all populations were found to be invariably C-band positive i.e. heterochromatic while the long arms were euchromatic. The telomere of long arms revealed prominent C-band positive staining. In some individuals of NXL and BDN the C-band was found to be localized at two distinct points of short arm of X, so that the short arm was differentiated into faint and darkly stained regions with strong C-band positive distal telomere (Fig. 1a, d). One female *Mus terricolor* in GDH population showed interesting result. One of the two X-chromosomes in this specimen was strongly stained at the telomeric end but the other X was totally devoid of C-band positive telomeric staining, while short arm was intensly C-band positive (Fig. 1c).

### Y chromosome

The entire Y chromosome was found to be consistently C-band positive in all populations; however, some differences were noticed in the intensity of banding (Table 2). Faintly stained Y chromosome was observed in NXL, GDH, KGM and MLB populations (Figs 1a, c, 2c and 3a), while rest of the populations revealed intensely stained Y which is the characteristic of the species. Like X chromosomes, the telomeric end of the Y was also found to be C-banded with population differences.

## Discussion

The mouse major satellite DNA, largely present as pericentromeric constitutive heterochromatin blocks in all chromosomes except Y, is highly repetitive (Jones 1970, Pardue and Gall 1970, Dev et al. 1973). This region has been found to be highly variable andfast evolving indicating its role in early stages of evolution (Shaw 1994). Constitutive heterochromatin has been shown to be highly polymorphic between and within species of *Mus* (Akeson and Davisson 1991, Forejt 1973, She 1990, Piálek 2005, Mitsainas et al. 2009). The studies on different rodents of the genera *Peromyscus*, *Mastomys*, *Oryzomys*, *Sigmodon*, *Rattus*, *Apodemus* and *Mus* revealed a common C-band pattern, i.e. large sized centromeric C-bands in the autosomes and X- chromosomes, and a completely heterochromatic small Y-chromosome (Modi 1987 and references there in).

*Mus terricolor* is an actively speciating incipient species complex in which constitutive heterochromatin is playing a major role in karyotype differentiation (Sen and Sharma 1983, Sharma et al. 1986, 1990, Bahadur 1995). Variation in autosomal C-positive heterochromatin in the range of populations studied, suggest that *Mus terricolor* is in a dynamic state of speciation. Variation and accumulation of heterochromatin have been shown in rodents by many workers. They have agreed that the accumulation of C-heterochromatin represents a recently evolved trait in rodents (Baverstock et al. 1976, Greenbaum and Baker 1978, Gamperl 1982a, Gamperl et al. 1982, Sen and Sharma 1983, Qumsiyeh et al. 1988, Gallardo 1991). C-band polymorphisms in terms of size variation in wild derived inbred strains of mice have also been reported by Akeson and Davisson (1991). In our study the presence of population specific and/ or chromosome specific large blocks of C-bands, either in homozygous or in heterozygous condition suggest an increase or accumulation of C-positive heterochromatin which is consistent with above findings.

C-band polymorphism in X chromosomes of *Mus terricolor* populations revealed interesting features. Two discrete heterochromatic blocks on short arms of X chromosomes in NXL and BDN (Fig. 1a, d) suggest segmental localization of heterochromatin. Balajee and Sharma (1994) have also shown the same result in *Mus terricolor* by digesting the metaphase chromosomes with *Alu* I and staining with Giemsa which produces C-band like features.

The large size of the Y chromosome in *Mus terricolor* is due to accumulation of C-positive heterochromatin (Sharma 1996) which shows population wise variation in banding intensity. In our study the cause of staining differentiation is not clear, though C-band polymorphism and apparent absence of C-positive chromatin in the Y chromosome has been shown in different species of rodents (*Phenacomys intermedius* Merriam, 1889, *Microtus californicus* Peale, 1848, *Microtus orchogaster* Schreber, 1842, *Clethrionomys californicus* Merriam, 1890 and *Microtus oregoni* Bachman, 1839, *Microtus arvalis* Pallas, 1778) by different workers (Zenzes and Voiculescu 1975, Gamperl 1982a, b, Vorontsov et al. 1984, Modi 1987) which suggests compositional heterogeneity of heterochromatin (Peacock et al. 1981, Patton and Sherwood 1982, Gallardo 1992) or unusual DNA sequences with different staining properties. (John and Miklos 1979, Peacock et al. 1981).

Populations of *Mus terricolor* showed prominent telomeric C-band on the long arm of X and also on acrocentric Y, but telomeric C-bands were not observed in autosomes in any population. Large prominent autosomal telomeric C-bands have been shown in the common wood mouse, *Sylvaemus sylvaticus* Linnaeus, 1758 by Nadjafova et al. (1993) and Nadjafova (2008) who implicated its role in differentiation of the species. The evolution of telomeric heterochromatin has been suggested to occur due to transposition and amplification of the centromeric satellite component (Hirning et al. 1989) in case of *Sylvaemus sylvaticus*. The situation in *Mus terricolor* needs to be intensively investigated for conclusive inferences.

Intra- and inter-specific karyotype evolution involving heterochromatin has been studied and discussed in many species but the evolutionary significance of heterochromatin is not established due to simultaneous involvement of chromosomal rearrangements, like inversions and Robertsonian translocations (Duffey 1972, Bradshaw and Hsu 1972, Pathak et al. 1973, Robbins and Baker 1981, Patton and Sherwood 1982, Davis et al. 1985, Modi 1987). However, evolutionary classification of the European wood mice of the subgenus *Sylvaemus* and genus *Apodemus* is based on chromosomal markers, like species specific C-positive heterochromatin (Orlov et al. 1996, Nadjafova 2008). Comparative FISH analysis of C–positive blocks of centromeric heterochromatin in different species of wood mice, *Sylvaemus* (Rubtsov et al. 2011) and three chromosomal forms of *Sylvaemus uralensis* Pallas, 1811 (Karamysheva et al. 2010) revealed variation in copy number and the level of homology of repetitive sequences as well as their localization. In the present study overall centromeric heterochromatin variation in size and intensity of bands in autosomes and heterochromosomes in *Mus terricolor* populations is also suggestive of quantitative as well as qualitative variation. Chatterjee et al. (2003) have commented on the basis of their southern hybridization experiments that *Mus terricolor* types differ in satellite DNA organization from that of *Mus musculus* Linnaeus, 1758, an allied species, and *Mus booduga*, the sibling species.

It can be concluded that very large to minute C-bands and even absence of C-bands in centromere of autosomes within and between populations of *Mus terricolor* indicates presence of differential amount of heterochromatin which might have evolved by non-reciprocal DNA turnover mechanisms in wild populations that has also been suggested by many workers (Dover 1982, Redi et al. 1990), however, this needs more extensive studies.
